# Serum from patients with ankylosing spondylitis can increase PPARD, fra-1, MMP7, OPG and RANKL expression in MG63 cells

**DOI:** 10.6061/clinics/2015(11)04

**Published:** 2015-11

**Authors:** Zaiying Hu, Dongfang Lin, Jun Qi, Minli Qiu, Qing Lv, Qiuxia Li, Zhiming Lin, Zetao Liao, Yunfeng Pan, Ou Jin, Yuqiong Wu, Jieruo Gu

**Affiliations:** The Third Affiliated Hospital of Sun Yat-Sen University, Department of Rheumatology, Guangzhou, China

**Keywords:** Ankylosing Spondylitis, Wnt Pathway, Gene Expression

## Abstract

**OBJECTIVES:**

To explore the effects of serum from patients with ankylosing spondylitis on the canonical Wnt/β-catenin pathway and to assess whether the serum has an osteogenic effect in MG63 cells.

**METHODS:**

MG63 cells were cultured with serum from 45 ankylosing spondylitis patients, 30 healthy controls, or 45 rheumatoid arthritis patients. The relative PPARD, fra-1, MMP7, OPG and RANKL mRNA levels were measured using quantitative real-time polymerase chain reaction. Associations between gene expression and patient demographics and clinical assessments were then analyzed.

**RESULTS:**

MG63 cells treated with serum from ankylosing spondylitis patients had higher PPARD, fra-1, MMP7 and OPG gene expression than did cells treated with serum from controls or rheumatoid arthritis patients (all *p*<0.05). RANKL expression was higher in MG63 cells treated with serum from patients with ankylosing spondylitis or rheumatoid arthritis than in those treated with serum from controls (both *p*<0.05). The OPG/RANKL ratio was also higher in MG63 cells treated with serum from ankylosing spondylitis patients than in those treated with serum from controls (*p*<0.05). No associations were found between the expression of the five genes and the patient demographics and clinical assessments (all *p*>0.05).

**CONCLUSIONS:**

: Serum from ankylosing spondylitis patients increases PPARD, fra-1, MMP7, OPG and RANKL expression and the OPG/RANKL ratio in MG63 cells; these effects may be due to the stimulatory effect of the serum on the Wnt pathway.

## INTRODUCTION

Ankylosing spondylitis (AS) is a chronic inflammatory disease characterized by new bone formation that progressively leads to ankylosis and functional disability. The inflammatory phase of AS shares similarities with other types of inflammatory arthritis such as rheumatoid arthritis (RA), with high levels of pro-inflammatory cytokine production and osteoclast activity and damage to the cortical bone of joints 1. However, after the inflammation subsides, excessive ossification occurs at the site of initial erosion. Inflammation is often viewed as the inciting cause of ossification 2,3. Controlling the inflammatory process, however, does not appear to prevent the development of ossifications in AS 4. How inflammation is initiated and how it progresses to bone formation and eventual ankylosis are poorly understood.

The Wnt signaling pathway plays critical roles in homeostasis and bone health in adults. It is involved in osteoblastogenesis and is regulated in part by inflammation 5-7. Many studies have suggested a role for the Wnt pathway in the process of new bone formation in AS, as it can provide a linkage between inflammation and ossification 8-12. In the canonical Wnt pathway, the interaction of Wnt proteins with their receptors leads to increased β-catenin levels and eventually to the expression of downstream genes 8. Serum from AS patients can increase β-catenin expression and these circulating bone formation-inducing β-catenin molecules are functionally prevalent in AS patients 8. However, whether the downstream genes of the canonical Wnt pathway can also be increased by serum from AS patients has not yet been reported.

Downstream targets of the canonical Wnt pathway include c-myc, c-jun, cycD, PPARD, fra-1 and MMP7. C-myc functions as a transcription factor that is associated with a variety of hematopoietic tumors 13, c-jun is involved in human malignancies 14 and cycD is another tumor-related gene 15. PPARD, which influences chondrocyte cell growth and differentiation 16, may affect height in adults through effects on osteoclast function 17. Fra-1 plays important roles in osteoblast differentiation and bone formation that might be important for the osteogenic differentiation stimulated by Wnt/β-catenin signaling 18 and MMP7 is involved in the breakdown of the extracellular matrix in normal physiological processes, as well as in disease states such as arthritis and metastasis 19. Because c-myc, c-jun and cycD are more related to tumors, whereas PPARD, fra-1 and MMP7 are considered in many studies to be involved in bone metabolism, we chose PPARD, fra-1 and MMP7 as the target genes to be detected.

Osteoprotegerin (OPG) is a protein secreted by osteoblasts that protects the skeleton from bone resorption via binding to receptor activator of NF-κB ligand (RANKL) 20. Wnt pathway activation can increase OPG expression 21. Whether serum from AS patients can affect OPG and RANKL expression and have an osteogenic effect through Wnt pathway regulation has not yet been reported.

In this study, we attempted to explore the effects of serum from AS patients on the canonical Wnt/β-catenin pathway by measuring the expression of its downstream target genes in human osteoblast-like MG63 cells. We also attempted to detect OPG and RANKL expression to assess whether AS serum has an osteogenic effect in MG63 cells.

## MATERIALS AND METHODS

### Patients

We recruited 45 patients who fulfilled the modified New York classification criteria for AS 22, 45 age- and sex-matched healthy subjects and 30 patients who met the American College of Rheumatology 1987 revised criteria for RA 23. The healthy controls were defined by the Nordic questionnaire 24. All patients were Chinese and were recruited from outpatient clinics at the Department of Rheumatology of the 3^rd^ Affiliated Hospital of Sun Yat-sen University. The study was approved by the local ethics committee and all participants gave written informed consent, in accordance with the Declaration of Helsinki.

Disease activity assessment was performed using the Bath AS Disease Activity Index (BASDAI) and the Bath AS Function Index (BASFI) in AS patients 25,26. Markers of inflammation (erythrocyte sedimentation rate [ESR] and C-reactive protein [CRP] levels) were measured in AS and RA patients. Serum samples were obtained from all participants and were stored in aliquots of 250 µl at -20°C. To avoid freeze-thawing, each experiment was performed with a different aliquot.

### Cell culture

Human MG63 osteoblasts obtained from the American Type Culture Collection (ATCC) were cultured in Dulbecco's modified Eagle's medium (DMEM, Gibco) supplemented with 10% fetal bovine serum (FBS, Gibco) and 1% penicillin/streptomycin in 6-well plates at a concentration of 2×10^5^/ml and were incubated in a humidified atmosphere of 5% CO_2_ at 37°C for 24 hours. After washing with phosphate-buffered saline (PBS), 250 µl of serum from either AS or RA patients or from normal subjects was added into each well and the cells were cultured with DMEM but without FBS for another 48 hours.

### Quantitative real-time PCR

After a total incubation period of 3 days 27, PPARD, fra-1, MMP7, OPG and RANKL expression in MG63 cells was measured. Total RNA was isolated using TRIzol reagent (Invitrogen) and 1 µg of total RNA was converted to cDNA using the PrimeScript^TM^ RT reagent kit (TaKaRa). Real-time PCR reactions were performed using SYBR Premix Ex^TM^ Taq II (TaKaRa) on an ABI PRISM 7000 Sequence Detection System (Applied Biosystems). The primers used are listed in Table 1. The following cycling program was used: 15 min preincubation at 95°C, 10 s denaturation at 95°C and 31 s annealing at 60°C for 40 cycles. To confirm the amplification specificity, the PCR products from each primer pair were subjected to melting curve analysis. Each reaction was amplified in triplicate and the threshold cycle (Ct) was calculated using the 2^−ΔΔCt^ method. Relative gene expression was normalized to GAPDH, used as an internal reference.

### Statistical analysis

Statistical analysis was performed using SPSS software. Variables were tested for normality by applying the Kolmogorov-Smirnov test. Data are presented as the mean ± standard deviation (SD) or as percentages, as appropriate. Correlations between gene expression and other variables were analyzed using Pearson's or Spearman's test, as appropriate. One-way analysis of variance (ANOVA) and t-tests were used for group comparisons. *P* values less than 0.05 (2-tailed) were considered significant.

## RESULTS

### Characteristics of the study subjects

The demographic and disease characteristics of the study subjects are shown in Table 2. No significant differences were found for sex and age between AS patients and healthy controls (both *p*>0.05). RA patients were older than AS patients and had shorter symptom durations (both *p*<0.05).

### Gene expression detected by quantitative real-time PCR

The relative PPARD, fra-1, MMP7, OPG and RANKL mRNA levels are listed in Table 3. The corresponding *p*-values for comparisons among the gene expressions in AS, RA and control serum-treated MG63 cells are listed in Table 4. When MG63 cells were cultured with AS serum, PPARD, fra-1, MMP7 and OPG expression was significantly higher than in cells treated with serum from RA patients or healthy controls (all *p*<0.05). No significant differences were found for PPARD, fra-1, MMP7 and OPG expression in MG63 cells treated with serum from RA patients and those treated with serum from healthy controls (all *p*>0.05). In cells cultured with AS or RA patient serum, RANKL expression was higher than that in cells cultured with healthy control serum (both *p*<0.05); however, the difference in RANKL expression in cells treated with AS and RA patient serum was not significant (*p*>0.05). The OPG/RANKL ratio was also higher in AS serum-treated cells (*p*<0.05), but no significant difference was found for that of RA-treated cells (*p*>0.05) compared to control serum-treated cells.

### Associations between gene expression and patient demographics and clinical assessments

The correlation coefficient (r value) and the corresponding *p*-value between the gene expression in AS-serum treated MG63 cells and patient demographics and clinical assessments are listed in Table 5. In AS patients, no associations were found between PPARD, fra-1, MMP7, OPG and RANKL expression and patient demographics and clinical assessments (all *p*>0.05).

## DISCUSSION

In this study, we measured the effects of serum from AS patients on human osteoblast-like MG63 cells by detecting the mRNA expression of downstream target genes of the canonical Wnt/β-catenin pathway. PPARD, fra-1, MMP7, OPG and RANKL expression and the OPG/RANKL ratio were significantly higher in AS serum-treated cells than in cells treated with serum from RA patients or healthy controls. We also found that such effects of AS patient serum were not correlated with age, symptom duration, the BASDAI, the BASFI, CRP levels or the ESR.

Previous studies have reported that AS serum-treated Jurkat T cells had higher active β-catenin levels compared with control serum-treated cells 8. Jurkat T cells are human peripheral blood leukemia T cells that have been widely used to explore the Wnt pathway in leukemia 28. MG63 cells, established as an osteoblastic cell line from a human osteosarcoma, are frequently used to study the mechanism of bone metabolism. Many studies have adopted MG63 cells as a model to investigate the Wnt pathway 27,29. The use of MG63 cells may be advantageous over Jurkat T cells for researching the role of the Wnt pathway in bone formation, as MG63 cells have more osteoblast-like characteristics; thus, the use of MG63 cells in this study makes our results more convincing.

The Wnt pathway is critically important for normal bone homeostasis, as the aberrant regulation of bone homeostasis has been suggested as a key element in the pathogenesis of AS 8,9. Dickkopf-1 (DKK-1) and sclerostin are the main inhibitory molecules that regulate the canonical Wnt pathway. The blockade of DKK-1 was shown to lead to the fusion of sacroiliac joints in an animal model of arthritis 10. Altered skeletal expression of sclerostin has also been linked to radiographic progression in AS 11. A number of studies have evaluated serum Dkk-1 and sclerostin levels in AS patients, but conflicting data have been reported 12,. The net effect of AS serum on the canonical Wnt pathway (suppression or promotion) remains inconsistent. Rather than focusing on the circulating concentrations of stimulatory or inhibitory molecules of the Wnt pathway in this study, we evaluated the effect of AS serum by measuring the expression of downstream genes of the Wnt pathway. We found that PPARD, fra-1 and MMP7 gene expression was increased in AS serum-treated MG63 cells. This finding supports the notion that the canonical Wnt pathway can be activated by serum from AS patients.

OPG and RANKL are important molecules in maintaining the balance of bone metabolism. The OPG/RANKL ratio increases during the differentiation of pre-osteoblastic cells into mature osteoblasts 20. Our results demonstrated that AS patient serum can increase OPG and RANKL expression and increase the OPG/RANKL ratio, which may eventually contribute to the formation of new bone. These effects may also be related to canonical Wnt pathway activation.

According to our data, PPARD, fra-1, MMP7, OPG and RANKL expression were not correlated with the BASDAI, the BASFI, CRP levels or the ESR in AS patients. The effects of inflammation on pathophysiological bone formation in AS remain contradictory. Despite significant clinical improvement, cytokine blocking strategies do not overcome new bone formation in AS, suggesting that the molecular processes eliciting syndesmophyte formation may differ from those of inflammation 4. In this study, the lack of correlation between the expression of downstream genes of the Wnt pathway and clinical assessments indicated that bone responses may not be tightly linked to inflammation in AS patients.

In conclusion, serum from AS patients can increase PPARD, fra-1, MMP7, OPG and RANKL expression and the OPG/RANKL ratio in MG63 cells. These effects may be due to the stimulatory effects of AS serum on the Wnt pathway.

## Figures and Tables

**Table 1:** Primers used in real-time PCR.

Gene	Forward primer sequence (5'-3')	Reverse primer sequence (5'-3')
PPARD	CTACGGTGTTCATGCATGTGAGG	GCACTTCTGGAAGCGGCAGTA
fra-1	GGAGGAAGGAACTGACCGACTTC	CTAGGCGCTCCTTCTGCTTCTG
MMP7	GCATGAGTGAGCTACAGTGGGAAC	CCACATCTGGGCTTCTGCATTA
OPG	TGGCACCAAAGTAAACGCAGAG	CTCGAAGGTGAGGTTAGCATGTC
RANKL	AAGATGGCACTCACTGCATTTATAG	TGATGTGCTGTGATCCAACGA
GAPDH	GCACCGTCAAGGCTGAGAAC	TGGTGAAGACGCCAGTGGA

**Table 2:** Demographic and clinical characteristics of the study subjects.

	AS patients	RA patients	Healthy controls
Male:female (% male)	39:6 (86.7)	5:25 (16.7)	39:6 (86.7)
Age (years)	28.8 ± 8.9	45.1 ± 12.4	28.9 ± 8.6
Symptom duration (years)	5.2 ± 4.4	3.8 ± 5.1	NA
HLA-B27 positive (%)	42 (93.3)	NA	NA
BASDAI	3.7 ± 4.2	NA	NA
BASFI	2.8 ± 3.6	NA	NA
CRP (mg/l)	21.3 ± 23.7	18.3 ± 22.1	NA
ESR (mm/h)	24.2 ± 20.7	27.2 ± 19.6	NA

Values are the mean ± standard deviation, unless otherwise stated. AS = ankylosing spondylitis; RA = rheumatoid arthritis; HLA-B27 = human leukocyte antigen B27; BASDAI = Bath Ankylosing Spondylitis Disease Activity Index; BASFI = Bath Ankylosing Spondylitis Functional Index; CRP = C-reactive protein (reference range <6 mg/L); ESR = erythrocyte sedimentation rate (reference range male <15 mm/h, female <20 mm/h); NA = not applicable.

**Table 3:** Relative PPARD, fra-1, MMP7, OPG and RANKL mRNA levels in MG63 cells cultured with serum from the study subjects.

	Serum of subjects		
Gene	AS patients	RA patients	Healthy controls
PPARD	1.36 ± 0.98	1.07 ± 0.67	1.00 ± 0.58
fra-1	1.16 ± 0.36	0.88 ± 0.34	1.00 ± 0.38
MMP7	1.89 ± 0.69	1.20 ± 0.95	1.00 ± 0.64
OPG	1.75 ± 1.12	1.15 ± 0.49	1.00 ± 0.54
RANKL	1.49 ± 1.00	1.42 ± 0.91	1.00 ± 0.51

Values are the mean ± standard deviation. AS = ankylosing spondylitis; RA = rheumatoid arthritis.

**Table 4:** *P* values for comparisons of gene expression among the ankylosing, rheumatoid arthritis and control serum-treated MG63 cells.

	Comparisons between sera of different subjects		
Gene	AS *vs*. control	AS *vs*. RA	RA *vs*. control
PPARD	0.035	0.028	0.234
fra-1	0.043	0.001	0.118
MMP7	0.000	0.028	0.279
OPG	0.000	0.002	0.315
RANKL	0.003	0.917	0.004

AS = ankylosing spondylitis; RA = rheumatoid arthritis.

**Table 5:** Associations between gene expression in ankylosing spondylitis-serum treated MG63 cells and patient demographics and clinical assessments.*

	PPARD	fra-1	MMP7	OPG	RANKL	OPG/RANKL
Age (years)	0.21,(0.80)	0.11,(0.49)	0.24,(0.55)	0.40,(0.59)	0.31,(0.79)	0.33,(0.44)
Symptom duration (years)	0.16,(0.75)	0.34,(0.36)	0.16,(0.79)	0.25,(0.75)	0.41,(0.68)	0.45,(0.39)
BASDAI	0.12,(0.92)	-0.31,(0.78)	0.16,(0.45)	-0.17,(0.54)	0.08,(0.26)	-0.23,(0.61)
BASFI	0.10,(0.56)	-0.19,(0.26)	0.37,(0.89)	-0.35,(0.83)	0.15,(0.47)	-0.65,(0.35)
CRP (mg/l)	0.02,(0.90)	-0.21,(0.18)	0.18,(0.25)	-0.10,(0.52)	0.00,(0.99)	-0.13,(0.41)
ESR (mm/h)	0.10,(0.50)	-0.17,(0.26)	0.13,(0.39)	-0.05,(0.75)	0.11,(0.48)	-0.15,(0.32)

**<?ENTCHAR ast?>:** Values are the r, (*p*) of correlation coefficient. AS = ankylosing spondylitis; BASDAI = Bath Ankylosing Spondylitis Disease Activity Index; BASFI = Bath Ankylosing Spondylitis Functional Index; CRP = C-reactive protein; ESR = erythrocyte sedimentation rate.
